# Thermography Research in Diabetic Foot: Insights From a Scopus-Based Bibliometric Study

**DOI:** 10.7759/cureus.62858

**Published:** 2024-06-21

**Authors:** Shukriah Aqilah Zakaria, Chooi Leng Low, Ren Yi Kow, Zamzuri Zakaria Mohamad, Mohd Radhwan Abidin, Aminudin Che Ahmad, Muhammad Wafiuddin Ahmad, Ahmad Hafiz Zulkifly, Ahmad Syahrizan Sulaiman

**Affiliations:** 1 Orthopedics, Traumatology, and Rehabilitation, International Islamic University Malaysia, Kuantan, MYS; 2 Radiology, International Islamic University Malaysia, Kuantan, MYS; 3 Mechanical and Automotive Engineering Technology, Universiti Malaysia Pahang Al-Sultan Abdullah, Pekan, MYS

**Keywords:** diabetic foot examination, thermal imaging, thermal imaging camera, diabetic foot disease, diabetic foot infection

## Abstract

Diabetic foot problems are among the most debilitating complications of diabetes mellitus. These problems incur significant economic costs and reduce quality of life. The integration of thermography technology in the screening and management of diabetic foot problems has been proven to be successful in recent years. By detecting changes in temperature, thermography helps identify early infections and assists in patient monitoring. These early successes have inspired more research and publications in this field. To date, a comprehensive bibliometric analysis of thermography-related research on diabetic foot using the Scopus database has not been conducted. This bibliometric analysis aims to fill this gap by reviewing the Scopus database from its inception until 2023 to examine the literature on thermography-related research on diabetic foot. A total of 342 articles met the selection criteria and were included in this analysis. The number of articles in this field remained low until the 2010s, when there was a sudden surge of interest that prompted numerous publications. Authors from the USA contributed the highest number of articles and had the greatest scholarly impact in this field. Despite the major contribution from the USA, there were numerous collaborations between various countries, underscoring the importance of international collaboration in advancing research and exchanging knowledge.

## Introduction and background

Diabetes mellitus is a profoundly debilitating non-communicable disease that has emerged as a significant public health problem [1-3]. Among its various macro-vascular and micro-vascular complications, diabetic foot infection is particularly notable [4-9]. Patients with diabetes mellitus are at a higher risk of experiencing foot problems, owing to peripheral neuropathy and compromised circulation, both of which are micro-vascular consequences of the disease [4-6]. Diabetic foot infections and their sequelae significantly contribute to illness and disability among diabetic patients [8,9]. Henceforth, it is crucial to identify patients with foot at risk and recognize patients with early signs of diabetic foot infection, so that early intervention can be performed to prevent lower limb amputations [4-7]. Previous studies have shown that temperature fluctuations in the lower limb can be associated with diabetic foot problems [10,11]. Building on this knowledge, thermography technology such as infrared thermography (IRT) has been proposed as part of the investigative tool in the management of diabetic foot problems [10-12]. In the era of the COVID-19 pandemic, this technology may potentially be useful in the evaluation of patients with diabetic foot problems [13-15].

IRT originated in the early 1800s when William Herschel discovered thermal radiation. Over the years, contributions from various researchers have developed IRT into a highly effective temperature measurement technique [11,12]. This technique is safe, non-invasive, and consistent, allowing for the swift assessment of radiating energy related to skin temperature. A healthy individual usually maintains a relatively stable core body temperature (within ±0.6°C), which is crucial for maintaining the body’s physiological process. In contrast, skin temperature can fluctuate considerably based on the body's thermoregulation needs [9-12]. Any object with a temperature above absolute zero (-273°C) emits electromagnetic radiation, known as infrared or thermal radiation [12]. An infrared camera detects, and measures emitted radiation, converting this data into a temperature reading. This process enables the identification of heat emanating from the body and generates images displaying distinct physiological thermal patterns. Capturing these images according to specific standards can potentially improve the quality of this technique in the future [9-12].

With the advancement in data mining, data collection has become more convenient. For instance, data harvested from Google Trends can be valuable for assessing public interest and predicting future trends [16,17]. Bibliometric analysis is a powerful tool for evaluating the scholarly impact of published literature [18-20]. It summarizes research interests, measures relationships and clustering within a field, and assesses academic quality and impact by tracking the number of publications and citations each article receives. Furthermore, it can unravel collaborations among authors, institutions, and countries, facilitating the formation of new collaborative projects with researchers of similar expertise. Moreover, bibliometric analysis can identify research gaps within a field, offering valuable insights and guidance to researchers and institutions [18-20].

## Review

Literature search and search strategy

**Figure 1:**
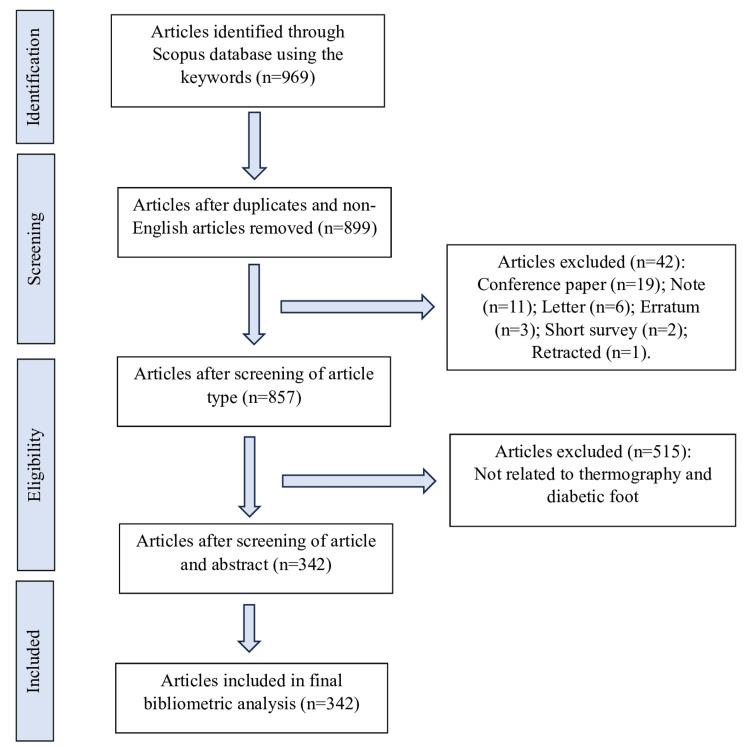
Flowchart of search results.

The literature on diabetic foot and thermography was sourced from the Scopus database. The search strategy was formulated using the following keywords (infrar* OR therm*) AND (diabet*) AND (foot*). The search spanned from January 1, 1961, to 31 December, 2023. The retrieved articles underwent screening by the two principal authors for suitability. Only English-language articles related to diabetic foot and thermography were included. Other types of publications such as conference papers, notes, letters, errata, short surveys, and retracted papers were excluded. Figure 1 summarizes the flow of the search and the search results.

The retrieved data were processed using R Studio 2021 for Windows (R Studio: Integrated Development for R, Boston, USA) with the “bibliometrix” package installed in R [21]. Information such as authorship, title, publication, number of citations, affiliations, journal source, references, and keywords were extracted using the R software. Data presentation, including illustrative graphs, was completed using “bibliometrix,” while Microsoft Excel 2019 was employed for data organization.

Results

Annual Scientific Production and Journal

**Figure 2:**
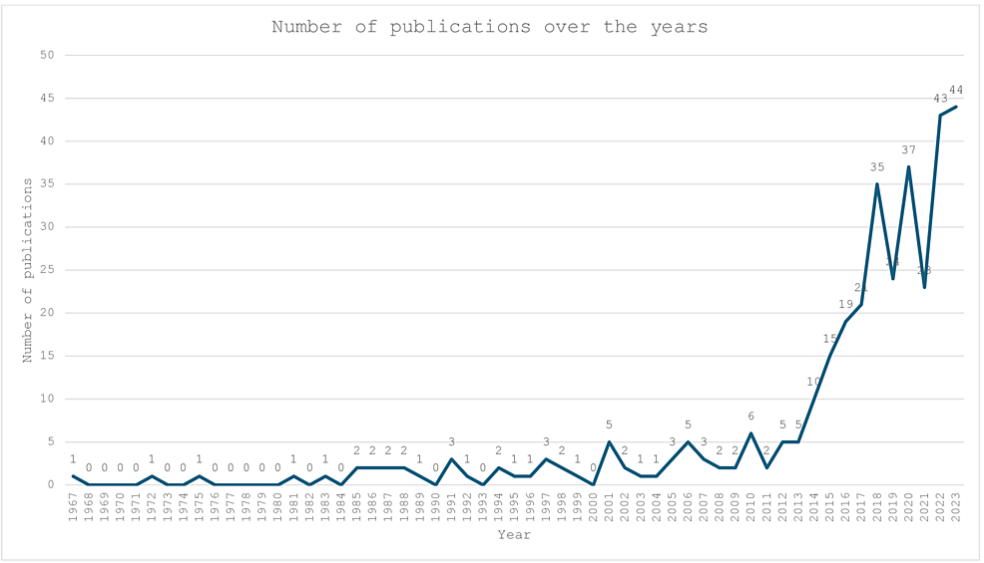
Number of publications over the years.

A total of 1093 articles were retrieved from the Scopus database from the years 1967 to 2023, using the terms presented. After screening by the lead authors, only 342 articles met the inclusion criteria and were analyzed in this review. As depicted in Figure 2, the first related article was published in 1967. There was very little publication activity in the late 1960s and early 1970s, with only occasional publications. The number of publications starts to increase more noticeably in the mid-1980s, with two or more publications per year. From 2013 onwards, the field experienced exponential growth in annual publications, with a total of 44 related articles published in 2023. Overall, the data suggests a trend of increasing publication activity over time, with some fluctuations from year to year. This could indicate continued interest and research in the topic, as well as potential new developments or areas of focus within the field.

A total of 342 articles were published in various journals within the study period. Table 1 presents the top 10 journals with the highest number of publications in the field of diabetic foot and thermography. The *Journal of Diabetes and Technology* has the highest number of articles, with 15. This is followed by *Infrared Physics and Technology* with 10 articles, and *Diabetic Medicine* with 8 articles. The *International Journal of Lower Extremity Wounds*, *Sensors*, and *Thermology International* each has seven articles, followed by *Diabetes Care*, *Journal of Wound Care*, *Proceedings of SPIE*, and *Proceedings of EMBC*, with each journal publishing six articles.

**Table 1:** Top 10 journals with the highest number of articles.

Journal	Number of articles
Journal of Diabetes and Technology	15
Infrared Physics and Technology	10
Diabetic Medicine	8
International Journal of Lower Extremity Wounds	7
Sensors	7
Thermology International	7
Diabetes Care	6
Journal of Wound Care	6
Proceedings of Spie - The International Society for Optical Engineering	6
Proceedings of The Annual International Conference of The IEEE Engineering in Medicine and Biology Society (EMBC)	6

Authors and Their Country of Origin

Table 2 summarizes the top 10 authors with the highest number of publications in the field of diabetic foot and thermography. Armstrong DG had the highest number of publications, with a total of 16 articles. This was followed by Peregrina-Barreto H (11 publications), van Netten JJ (11 publications), Bus Sa (11 publications), Ammer K (9 publications), Harba R (9 publications), Lavery LA (9 publications), Oe M (8 publications), Sanada H (8 publications), and Canals R (7 publications).

**Table 2:** Top 10 authors with the highest number of publications.

Author’s name	Number of articles
Armstrong DG	16
Peregrina-Barreto H	11
van Netten JJ	11
Bus Sa	10
Ammer K	9
Harba R	9
Lavery LA	9
Oe M	8
Sanada H	8
Canals R	7

Table 3 illustrates the top 10 countries with the highest number of publications in the field of diabetic foot and thermography. The USA leads in the number of publications in this field, with a total of 31 articles attributed to authors from the USA. This is followed by India (23 publications), China (16 publications), Mexico (15 publications), Portugal (15 publications), Spain (14 publications), the United Kingdom (11 publications), Brazil (9 publications), the Netherlands (9 publications), and Japan (8 publications). Among the top 10 countries, seven of them had a multiple-country production (MCP) ratio of more than 0.2 (20%). Up to 85% of the articles published by authors from India had collaborated with another country.

**Table 3:** Corresponding author’s country with the most number of publications. SCP: single country production; MCP: multiple country production

Country	Number of articles	SCP	MCP	MCP ratio
USA	31	27	19	0.129
India	23	20	4	0.130
China	16	13	3	0.188
Mexico	15	15	0	0.000
Portugal	15	9	6	0.400
Spain	14	14	0	0.000
United Kingdom	11	9	2	0.182
Brazil	9	9	0	0.000
Netherland	9	5	4	0.444
Japan	7	6	1	0.143

Types of Articles

Regarding the types of articles, the majority of the articles, totaling 210, fall into the clinical category, comprising 62% of the total. This is followed by non-clinical articles, accounting for 21% of the total. There are 42 review articles, making up 12% of the total. Thirteen articles are case reports, accounting for 4% of the total. There are four articles categorized as book chapters, representing 1% of the total. Table 4 summarizes the types of all articles reviewed in this bibliometric analysis. Overall, the data shows a diverse range of article types, with the majority of the articles being clinical in nature, followed by non-clinical and review articles.

**Table 4:** Categorization of the articles.

Type of articles	Number of articles	Percentage
Non-clinical	73	21
Clinical	210	62
Review	42	12
Case report	13	4
Book chapter	4	1
Total	342	100

Top 10 Articles With the Highest Number of Citations

Table 5 shows the top ten articles with the most citations within the study period. The publication titled "Preventing diabetic foot ulcer recurrence in high-risk patients: use of temperature monitoring as a self-assessment tool" received the highest number of citations (321), by Lavery from the USA [22]. Another highly cited publication (298 citations) by Armstrong et al. discusses how skin temperature monitoring reduces the risk of diabetic foot ulceration in high-risk patients. It was published in 2007 in the *American Journal of Medicine* [23]. This was followed by “Home monitoring of foot skin temperatures to prevent ulceration” by Lavery (275 citations), “Evaluation of thermal and vibration sensation in diabetic neuropathy” by Guy (191 citations), “Infrared dermal thermometry for the high-risk diabetic foot” by Armstrong (164 citations), “Correlation between plantar foot temperature and diabetic neuropathy: A case study by using an infrared thermal imaging technique” by Bagavathiappan (140 citations), “Thermography and thermometry in the assessment of diabetic neuropathic foot: A case for furthering the role of thermal techniques” by Bharara (123 citations), “Monitoring healing of acute Charcot’s arthropathy with infrared dermal thermometry” by Armstrong (120 citations), “Methods of measurement of thermal thresholds” by Claus (118 citations) and lastly in the top 10 the publication by Liu, “Automatic detection of diabetic foot complications with IRT by asymmetric analysis” (104 citations) [14-31].

**Table 5:** Top 10 articles with the highest number of citations.

Number	Citation	Citation per year	First author	Country	Title	Year	Journal	Type
1.	321	17.83	Lavery et al. [22]	USA	Preventing diabetic foot ulcer recurrence in high-risk patients: use of temperature monitoring as a self-assessment tool	2007	Diabetes Care	Clinical
2	298	16.56	Armstrong et al. [23]	USA	Skin temperature monitoring reduces the risk for diabetic foot ulceration in high-risk patients	2007	American Journal of Medicine	Clinical
3	275	13.10	Lavery et al. [24]	USA	Home monitoring of foot skin temperatures to prevent ulceration	2004	Diabetes Care	Clinical
4	191	4.78	Guy et al. [25]	UK	Evaluation of thermal and vibration sensation in diabetic neuropathy	1985	Diabetologia	Clinical
5	164	5.86	Armstrong et al. [26]	USA	Infrared dermal thermometry for the high-risk diabetic foot	1997	Physical Therapy	Clinical
6	140	9.33	Bagavathiappan et al. [27]	India	Correlation between plantar foot temperature and diabetic neuropathy: a case study by using an infrared thermal imaging technique	2010	Journal of Diabetes Science and Technology	Clinical
7	123	6.47	Bharara et al. [28]	United Kingdom	Thermography and thermometry in the assessment of diabetic neuropathic foot: a case for furthering the role of thermal techniques	2006	International Journal of Lower Extremity Wounds	Clinical
8	120	4.29	Armstrong et al. [29]	USA	Monitoring healing of acute Charcot’s arthropathy with infrared dermal thermometry	1997	Journal of Rehabilitation Research and Development	Clinical
9	118	3.11	Claus et al. [30]	Germany	Methods of measurement of thermal thresholds	1987	Acta Neurologica Scandinavica	Clinical
10	104	10.40	Liu et al. [31]	Netherland	Automatic detection of diabetic foot complications with infrared thermography by asymmetric analysis	2015	Journal of Biomedical Optics	Clinical

Collaboration Between Different Countries

**Figure 3:**
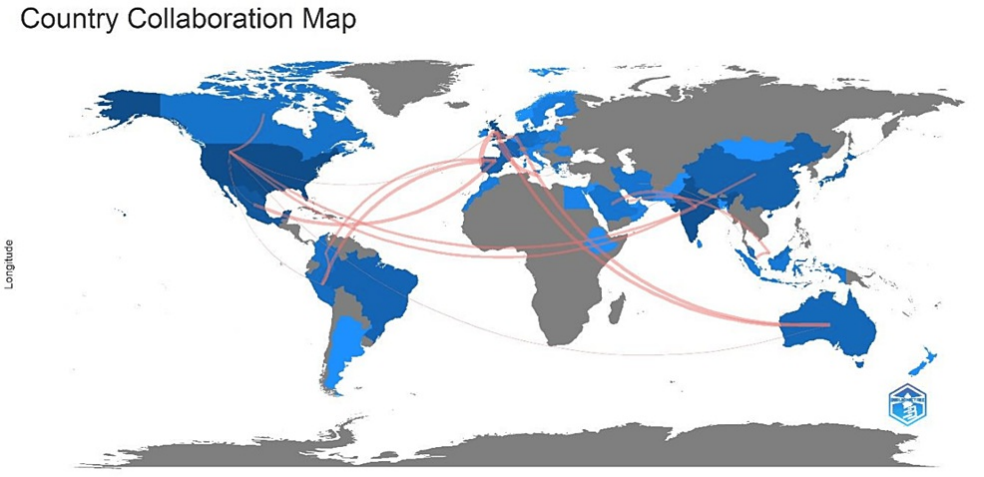
Countries' collaboration world map. The pink lines depict the collaboration between countries with the thickness of the pink line indicating the degree of collaboration. Note: Figure generated using the bibliometrix software in R Studio (R Studio: Integrated Development for R, Boston, USA) [21]

Authors from different countries engage in collaborative research and partnerships with researchers from various other countries (Figure 3). The authors from the USA collaborate with researchers from Canada, China, India (three publications), Mexico, Australia, and Peru (two publications), as well as one publication each from Iran, Japan, Qatar, Brazil, and Singapore. Meanwhile, the authors from India collaborate with researchers from Brazil, Ethiopia, Malaysia, Oman, Pakistan, and Saudi Arabia. This diverse, global, and interconnected approach underscores the importance of international collaboration in advancing research and exchanging knowledge.

Discussion

The Scopus database published by Elsevier, is an extensive collection that includes a wide range of journals, conference proceedings, and patents [18-20]. Scopus is a multidisciplinary database. It indexes documents from sciences, social sciences, arts, and humanities. In addition to providing basic information like authors' details and abstracts, Scopus offers comprehensive citation analysis, including metrics such as the h-index and citation counts [18]. As the academic environment progresses, it becomes increasingly important to grasp the intricacies of each database, as they each possess unique strengths and limitations. Keeping this in consideration, this study conducts a bibliometric analysis to explore the utilization of thermography in diabetic foot ulcers using the Scopus database. This review confirms a broad array of important details regarding the authors, subjects, and time frames that have significantly influenced the management of diabetic foot issues.

In this review, we highlight 10 articles with the highest number of citations in the Scopus database that are relevant to diabetic foot ulcers and thermography. Although citation analysis has its limitations, it offers an impartial method for gauging peer acknowledgment and provides valuable insights into the audience of scholarly articles [18-20]. It is crucial for researchers to acknowledge that citation rates alone may not fully capture the multifaceted nature of research impact. While not directly linked to study quality, the high citation counts indicate the impact and relevance of these studies within the scientific and medical communities. Based on the top three articles, it is evident that there is a growing emphasis on preventive measures such as skin temperature monitoring to identify early signs of complications. These studies demonstrate that regular monitoring can effectively reduce the risk of ulceration, emphasizing prevention as a critical aspect of diabetic foot care. The majority of the highly cited articles are clinical studies focusing on practical and applicable outcomes for patient care. Examples include studies on skin temperature monitoring, triaxial stresses, and the use of infrared thermometry. This highlights the field's focus on direct patient care and the implementation of research findings to enhance clinical practices.

While the majority of highly cited articles come from the USA, there is also a significant contribution from other countries, such as the UK, Germany, Netherlands, and India. This highlights the global effort in addressing diabetic foot complications and the collaborative nature of this research field. There is also a notable international collaboration and contribution to the field, highlighting its global importance.

The USA's frequent presence on the list could be attributed to several factors, such as its robust research infrastructure, which includes universities, hospitals, and research institutions, potentially enabling higher research output. Furthermore, the USA, the UK, the Netherlands, and Germany are developed countries with well-established research funding systems that provide numerous grants for medical research. This could result in a higher volume of research being carried out and published from these regions.

The inherent nature of selection bias also contributes, as these countries predominantly use English for scientific communication. Similarly, English-language articles often reach a broader global audience, leading to increased citation rates.

Limitations

There are several limitations in this bibliometric analysis. First, this review exhibits selection bias, as it includes only English articles derived from the Scopus database. Therefore, the dataset may be biased toward certain journals, languages, or regions as it includes only English publications or journals. Third, the analysis may be influenced by the time frame of the literature search. The number of citations received by the articles reviewed in this bibliometric analysis may be potentially higher, particularly for articles published in recent years. Furthermore, the citation count may change if additional databases - like Web of Science Core Collection or Google Scholar - are used, as this bibliometric analysis solely looks at the Scopus database. With the presence of these intrinsic limitations, this bibliometric analysis may not paint a complete picture of the research in this field.

## Conclusions

Overall, research on the diabetic foot and thermography showcases a field marked by growing research activity, technological progress, and a significant clinical emphasis. This review provides an in-depth overview and bibliometric analysis of publications on diabetic foot and thermography from the Scopus database, spanning various time periods. The significant growth in publications and high-impact studies underscores the importance of thermography as a valuable tool for the early detection, prevention, and management of diabetic foot complications. Moving forward, continued global collaboration, technological innovation, and emphasis on preventive strategies will be crucial in advancing the field and improving patient outcomes.
